# An Anecdotal Case Report of Chronic Lymphatic Leukemia with del(11q) Treated with Ibrutinib: Artificial Nourishment and Physical Activity Program

**DOI:** 10.3390/ijerph17061929

**Published:** 2020-03-16

**Authors:** Antonello Sica, Caterina Sagnelli, Alfonso Papa, Massimo Ciccozzi, Evangelista Sagnelli, Armando Calogero, Erika Martinelli, Beniamino Casale

**Affiliations:** 1 Department of Precision Medicine, University of Campania Luigi Vanvitelli, 80131 Naples, Italy; erika.martinelli@unicampania.it; 2 Department of Mental Health and Public Medicine, University of Campania Luigi Vanvitelli, 80131 Naples, Italy; sagnelli.caterina@libero.it (C.S.); evangelista.sagnelli@unicampania.it (E.S.); 3 Pain Department, AORN Dei Colli V. Monaldi, 80131 Naples, Italy; alfonsopapa@libero.it; 4 Medical Statistics and Molecular Epidemiology, Campus Bio-Medico University, 00100 Rome, Italy; m.ciccozzi@unicampus.it; 5 Department of Advanced Biomedical Sciences, University of Naples Federico II, 80131 Naples, Italy; armando.calogero2@unina.it; 6 Department of Pneumology and Tisiology, AORN Dei Colli - V. Monaldi, 80131 Naples, Italy; bennycasale@hotmail.com

**Keywords:** physical activity, elder age, CLL, del(11q), ibrutinib

## Abstract

Chronic lymphatic leukemia (CLL) is the most frequent type of leukemia in western countries and when association with del(11q) is correlated with a worse prognosis. We reported the clinical case of an 80-year-old patient with CLL related to del(11q) and a BMI of 16.4 kg/m^2^, who presented a voluminous mass in abdominal cavity (23 × 14 × 4 cm) which occupied the whole of the mesentery and the retroperitoneal space, treated with ibrutinib, adequate nutrition, and a program of physical activity. He showed a great improvement under ibrutinib therapy and took to artificial nourishment and adequate muscle rehabilitation until he recovered his autonomy. In August 2018, a 5-days-a-week training program was started: Physical activity for at least 20 min consisting of a fast walk in the open air three times a week and a moderate physical activity in the remaining two days of at least 20 consecutive minutes (cycling at a regular pace, carrying light weights). The exercise program included also aerobic, upper and lower limb resistance training, chore stability and stretches. The physical condition further improved and remained excellent throughout the follow-up period. In December 2018, his clinical condition was quite normal; a CT showed a great decrease of all lymphoadenomegaly, and FISH test did not show del(11q). He continued to cultivate his land, while still being treated with ibrutinib. The combination of the right therapy, adequate nutrition, and muscle rehabilitation is the best solution to improve the clinical condition of old cachectic CLL del(11q) patient.

## 1. Introduction

Chronic lymphatic leukemia (CLL) is the most frequent type of leukemia in western countries, and in patients >65 years-of-age (70% of cases) with a median age of 72 years. CLL incidence rate is around 4/100,000 inhabitants per year [1], with an increase to more than 30/100,000 in elderly subjects (>80 years) [2]. The CLL associated with del(11q) or del (17p) among clinical forms with the worst prognosis [3]. Clinical studies have shown that Ibrutinib has been used to treat the clinical form associated with the mutation del(17p), a more favorable response than that obtained with a conventional chemotherapy (rituximab, cyclophosphamide, fludarabine) [4]. This is the first clinical report of an 80-year-old patient with CLL related to del(11q), who presented voluminous solid tissue in the abdominal cavity (23 × 14 × 4 cm) which occupied the whole of the mesentery and the retroperitoneal space by displacing the pancreas anteriorly and the intestinal loops laterally, which prevented adequate nutrition. In cases like this, despite the possibility of an effective therapy being assigned, there is a point of no return where recovery may not be achieved. The recovery of muscle function in cachectic patients is fundamental, particularly in elderly patients who may develop a depressive psychological syndrome, certain they cannot cope the evolution of the disease [5,6]. In these clinical cases, active and passive motor rehabilitation is necessary to maintain a muscle tone to favor an upright position and, whenever possible, walking capacity [7,8]. Subsequently, patients should be helped and then more in small steps or remained in an upright position with prolonged support [9,10]. The 2017 guidelines of the Italian Association of Medical Oncology (AIOM) recommend physical activity in neoplastic cachexia. It allows stabilization and recovery of muscle mass, promotes protein synthesis, reduces inflammatory response, and improves antioxidant activity, insulin resistance, immune status, cardiorespiratory and muscular fitness, and bone health [11,12,13,14,15]. Furthermore, physical activity might have a favorable impact on quality of life in elderly cancer survivors, increasing their physical abilities, avoiding depression, increasing their self-esteem, reducing the risk of cognitive decline, and promoting relaxation [14,15,16,17]. Once the recovery of autonomy has been obtained, an out progressive physical activity should start according to the patient’s physical abilities [1,11]. It is advisable to start with long walks, to increase progressively in rhythm and duration, accompanied by aerobic, upper and lower limb resistance training, and stretches [12]. Few data are available in the exercise program management in oncological and hematological setting and the lack of criteria for starting motor “muscle” rehabilitation, in particular in cachectic and elderly patients is reported [12,13,14,15,16,17,18,19,20,21]. Walter et al. evaluated in the prospective VITamins and Lifestyle (VITAL) study on 364,418 oncological patients, aged 50–76 years, the impact of physical activity on hematologic malignancies; and observed the decreased risk of hematologic cancer associated with physical activity, in myeloid neoplasms, CLL/small lymphocytic lymphoma, and in other mature B-cell lymphomas [22].

In our case, after a cardiological evaluation, when he had regained his autonomy, the patient was encouraged to intensify the physical activity he carried out progressively, avoiding excesses that caused palpitations or dyspnea to occur. After this step, it was easier to make use of the previously-mentioned physical activity programs modified for our patient’s ability, attitudes, clinical, and social conditions. The ultimate goal was to bring the patient back to the type of activity he did before the disease.

This paper describes the first clinical case of a patient with little chance of survival treated with ibrutinib and underwent a program of physical activity. The aim of our report is to underline the importance of rehabilitation which, associated with the pharmacological treatment was of great help for 80-year old CLL del(11q) patient.

## 2. Case Report

**Figure 1:**
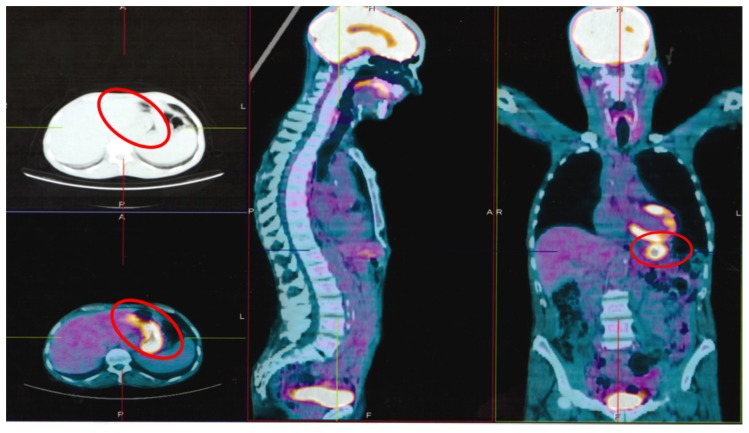
[18F] FDG-PET performed with co-registered CT at the diagnosis (February 2018) show significant areas (indicated in red circles) of increased metabolic activity in abdominal cavity loaded with solid tissue (23 × 14 × 4 cm) which occupies the whole of the mesentery and the retroperitoneal space by displacing the pancreas anteriorly and the intestinal loops laterally (SUV 4.85).

**Figure 2:**
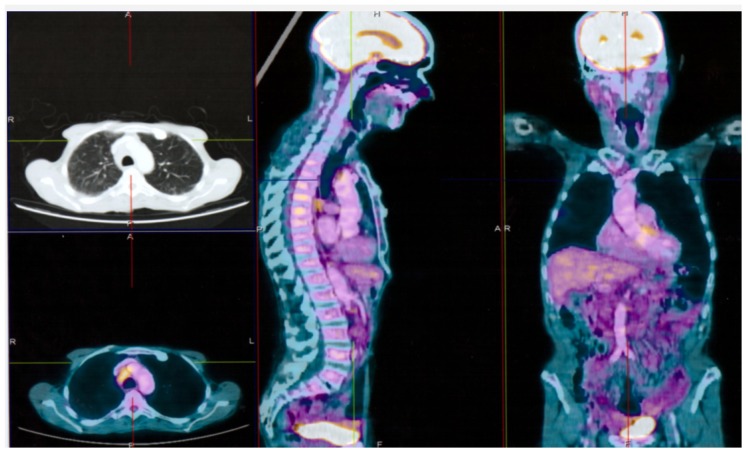
[18F] FDG-PET performed with co-registered CT in follow up (October 2019) not show significant areas of increased metabolic activity in abdominal cavity.

An 80-year-old Caucasian man was observed at our clinical center in January 2018, for unintentional weight loss of 5 kg, itching without any skin lesion [23,24], and stomachache. His blood cell count showed lymphocytosis. No noteworthy pathologies were reported in the anamnesis. He was still active in cultivating his soil, but recently he was less active. [18F] FDG-PET performed with co-registered CT in February 2017 (Figure 1) showed a significant area of increased metabolic activity in abdominal cavity loaded with solid tissue (23 × 14 × 4 cm) which occupies the whole of the mesentery and the retroperitoneal space by displacing the pancreas anteriorly and the intestinal loops laterally (SUV 4.85) and splenomegaly [25]. The bone marrow examination showed a 30% infiltration of phenotypically clonal lymphocytes k+, CD20 +, CD5 + CD23 + CD10−, with del (11q) in FISH test with evidence of an un-mutated immunoglobulin heavy-chain variable region (IGHV) gene. The patient was affected by CLL stage II according to Rai with symptomatic active bulky disease and he needed treatment. The patient was also tested for serum markers of HIV, HBV, HCV, and CMV and was found positive only for anti-HBc, as a sign of previous HBV infection [26,27,28,29,30]. Lamivudine prophylaxis at the dose of 100 mg/day was administered 4 weeks before the start of chemotherapy, in order to prevent HBV reactivation. In March 2017, chlorambucil therapy was administered together with oral opioids for pain control [31]. In the following 2 months, the patient lost other 5 kg, because of the difficulty in feeding, and suffered of severe fatigue, which forced him to bed all day long; at this time, his body weight was 40 kg and his BMI 16.4 kg/m^2^. On 2 May 2018, he performed all blood chemistry tests that showed the following anomalies: Erythrocyte sedimentation rate (ESR) 71 mm; C-reactive protein (CRP) 54 mg/L; fibrinogen 637 ng/dL, lactate dehydrogenase (LDH) 498 U/L; albumin 2.6 g/dL; triglyceride, cholesterol and glicemy levels were at the lower limits of normal; a moderate anemia with hemoglobin 9.5 g/dL, a lymphocytosis (20,000/microliter). At cardiology consultation and echocardiogram he showed good functionality. Chemotherapy was changed and ibrutinib 420 mg/day was introduced in June 2017. In addition, a rehabilitation physio kinesitherapy program and parenteral nutrition were started associated with intravenous (i.v.) albumin every day and, erythropoiesis-stimulating agents (ESAs) subcutaneous (s.c.) every week. Peripheral parenteral nutrition included the intake of glucose, lipids, branched chain amino acids, B vitamins, and folate. The muscle exercise program was based on an attempt to bring the patient back to physical capacity 4 months before bedtime. After one month, the general conditions began to improve. He started walking with support and gradually he was able to walk and eat independently. His performance status improved progressively, and he was recommended to walk for 30-minutes a day at mild intensity in July 2018 [32]. His CT showed an important decrease of all lymphoadenomegaly, and his blood count become normal, with ESR 40 mm; CRP 20 mg/L. In August 2018, a 5-days-a-week training program favoring some hobbies of the patient was started: A physical activity for at least 20 min consisting of a fast walk in the open air three times a week and a moderate physical activity in the remaining two days of at least 20 consecutive minutes (cycling at a regular pace, carrying light weights) [33,34,35]. The exercise program included also aerobic, upper and lower limb resistance training, core stability, and stretches [10,36,37]. Appetite increased more and more along with his ability to feed and digest all foods. The aerobic training was first increased to 30–40 min walking or cycling stationary at moderate and progressive intensity and after to 60 min. The physical condition further improved and remained excellent throughout the follow-up period. All blood tests progressively improved; in October 2018, they showed only a slight increase in pancreatic amylase (77 U/L) and lipase (83 U/L). In December 2018 his clinical condition was quite normal, a CT showed a great decrease of all lymphoadenomegaly, FISH test did not show del(11q) and all blood tests are normal. Now, the patient is in good clinical condition and thanks to the physical training program, he has achieved an enviable physical condition. He continued to cultivate his land, and is still being treated with ibrutinib; his last CT performed in October 2019 showed good partial response (Figure 2).

## 3. Discussion

The diagnosis and management of CLL impact quality of life (QoL) [37], and also the sedentary lifestyle of the majority of cancer patients play a negative effect on QoL [5,6,37,38,39,40,41,42] and on their survival [11]. However, randomized studies have shown the substantial improvement in QoL of cancer patients due to an adequate program of physical activity [23,24,25,34] and impact the cancer survival [7], since it reduces the peak oxygen consumption, improves physical capacity, increases self-esteem, reduces accumulated stress, and promotes relaxation [8,11]. Consequently, physical activity is strongly recommended for healthy individuals to improve cardiorespiratory and muscular fitness, bone health, and in general to reduce the risk of non-communicable diseases, depression, and cognitive decline [11] The correct application of an exercise program may have a beneficial effect in onco-hematologic patients, since it may improve the function of the immune system (regulation of macrophages and natural killer cells interacting with cancer cells) [9,10] and provide a protective effect against cancer progression. In our clinical center, we encourage cancer patients, to progressively increase their physical activity, depending on their abilities and attitudes, according to the Cancer Patient Support Organizations such as the Lymphoma Action and includes walks, planned exercises, sports activities, domestic activities, and professional activities [26]. The type of program is different from one patient to another. The first step is a functional recovery and autonomy that is usually carried out in hospital with physiokinesi therapy and psychological support [5]. Once discharged, the patients should start a psychophysical rehabilitation program aimed to improve the functional capacity the behavioral conditions and the quality of life (QoL). The preliminary evaluation is multidimensional and should include general clinical conditions, functional state of different organs, psychic state, physical-motor capacity (strength, flexibility, and muscular coordination), cardiovascular function under stress conditions, comorbidities and social conditions [6,7]. Training can produce extremely important effects, as older people use a considerable proportion of their functional capacity to perform even simple everyday activities [8]. The relationship between physician and patient is a more important support to improve the patient’s QoL. Physicians can assist patients in the acceptance and perception of cancer disease by improving their knowledge with information in according with the educational level. In fact, doctors, based on the needs of the patients, can stimulate improvement of physical activity, nutritional behavior, and social and emotional status of patients and also in the relationship between patient and their family members [37,38].

The associated physical activity program allowed our patient to reach a good shape and to remain so for all the observation period [43,44,45,46,47,48]. We also believe that the advice to practice physical activity has generated optimism in our patient suggesting that the illness is a moment of passage to be faced and overcome [43,44,45,46,47,48].

## 4. Conclusions

The CLL with del(11q), usually show a poor prognosis and a very old patient is not the ideal candidate to respond to treatment. According to the international Prognostic Score (CLL-IPI), our patient was to be considered in very high-risk with a 5-year overall survival (OS) of about 23%. However, the new tyrosine kinase inhibitors (TKI) allow the older patients who usually present comorbidities and poor physical condition because they are more tolerable than conventional chemotherapy [29]. However, the increased therapeutic possibilities in elderly patient is not a guarantee of therapeutic success, since a particularly severe clinical condition may represent a point of no return [30]. Over 70% of patients with cancer, especially in the advanced stages, develop cachexia and about 20% die of it. Often, as in our case, this syndrome is secondary to a mechanical obstruction created by the bulky mass on the digestive tract, which makes feeding impossible. The advanced stages of this syndrome make the prognosis extremely severe and irreversible, therefore in the early stages it is essential that muscle recovery occur as soon as possible. In the absence of an adequate nutritional intake, recovery is impossible. In the first phase, in our case, specific therapy of neoplastic disease and parenteral nutrition played a key role, while motor rehabilitation allowed the stabilization of muscle mass. Subsequently, once the patient responded to Ibrutinib and began to benefit from the reduction of the neoplastic masses and therefore to be able to physiologically provide the nutrients necessary for the body’s needs, the role of physical activity was equally crucial in bringing the patient progressively back to autonomy and providing him with the stimuli and self-esteem to overcome this critical moment. Without the action of cancer therapy, the patient would not have passed this phase, but parenteral nutrition and muscle recovery also played a fundamental role in different phases, without which it would have been extremely difficult.

Associated to treatment, adequate nutrition, muscle rehabilitation and psychological support allow a better chance of recovery, our patient in on reliable example of this [23,24].
